# Effects of manual therapy in addition to stretching and strengthening exercises to improve scapular range of motion, functional capacity and pain in patients with shoulder impingement syndrome: a randomized controlled trial

**DOI:** 10.1186/s12891-024-07294-4

**Published:** 2024-03-02

**Authors:** Sana Tauqeer, Ayesha Arooj, Hammad Shakeel

**Affiliations:** University Institute of Physical Therapy, University of Lahore, Lahore, Pakistan

**Keywords:** Functional capacity, Pain, Range of motion, Shoulder impingement syndrome

## Abstract

**Background:**

The current study aimed to measure the effectiveness of manual therapy in addition to stretching and strengthening exercises in patients with shoulder impingement syndrome to improve functional capacity, pain, and scapular range of motion.

**Methods:**

This is a single-blinded randomized controlled trial. Thirty-two participants with chronic shoulder impingement syndrome were randomly allocated into two groups. Both groups received stretching and strengthening exercises while the treatment group was given manual therapy additionally. Treatment was started after the patients signed an informed consent form. The data were collected from the University of Lahore Teaching Hospital between March 2022 and December 2022. The study aimed to measure pain using a numeric pain rating scale, functional capacity was assessed by the disability of the arm and shoulder, and goniometry was used for scapular ranges, i.e., scapular protraction and upward rotation. Each treatment session lasted 45 min for the treatment group and 30 min for the control group. The treatment comprised five days a week for four weeks, after which post-intervention measurements were taken.

**Results:**

Thirty-two participants were enrolled in the study, and 16 were divided into each group. The mean age of the participants in the treatment group was 38.19 ± 7.31 while the comparison group was 35.69 ± 7.98. An independent sample t-test was run on the data with a 95% confidence interval, statistically significant results were obtained, i.e., p-value < 0.05, post-intervention in the treatment group. Both groups have significantly improved functional capacity and scapular protraction (*p* < 0.005), however, pain and scapular upward rotation were not found statistically significant in the control group (*p* > 0.05).

**Conclusion:**

The addition of manual therapy along with exercise therapy showed clinical and statistical significant results for pain, functional capacity, and scapular range of motion. It demonstrated superior effects than exercise therapy alone for the chronic condition of SIS.

**Trial Registration:**

The trial was registered in the Iranian Registry of Clinical Trials (https://www.irct.ir/) with the registration number: IRCT20230526058291N1, (Date: 12/08/2023).

**Supplementary Information:**

The online version contains supplementary material available at 10.1186/s12891-024-07294-4.

## Introduction

Shoulder impingement syndrome (SIS) is a common cause of musculoskeletal-originated shoulder pain, which is associated with repetitive work above or at the level of the shoulder [1]. Additionally, it has also been commonly seen in athletic populations who use overhead activities [2, 3]. Most reasons that came under consideration for shoulder pain have a musculoskeletal basis, in which most of the population has prevalent SIS [3, 4]. According to a recent study, the estimated prevalence of shoulder pain ranges between 7% and 34% in which the highest complaint is recorded with the etiology of SIS [5]. A study indicated global incidence for SIS as 44–65% [6]. Moreover, another study accounted for a relatively high prevalence of shoulder pain in the working population of Pakistan [7, 8]. SIS has been reported highly common disorder in Pakistan [7]. According to a study conducted in early 2023, 49% of prevalence has been demonstrated in the athletic population of Pakistan [9]. 

It is important to identify shoulder impingement because if left untreated it may end up in degenerative changes at the shoulder joint, [10] chronic pain, and, sometimes more severe conditions such as rotator cuff tears [11]. Shoulder impingement can be demonstrated following the location of impingement as external and internal impingement or underlying cause may be primary or secondary impingement [12–14]. A mechanical or physical intrusion of the soft tissue inside the sub-acromial space causes an external impingement, also known as sub-acromial impingement [13]. On the other hand, internal impingement develops when the rotator cuff tendons invade the space between the glenoid rim and the humeral head. Most frequently, internal impingement is associated with the tendons of the supraspinatus and infraspinatus [12]. Primary impingement is the structural narrowing of sub-acromial space due to direct trauma, abnormal anatomy of acromion space, or swelling in soft tissues [12]. Secondary impingement occurs due to abnormality in the surrounding structures such as weakness of trapezius or serratus anterior muscles, rotator cuff weakness, or uncontrolled translation of the humeral head [15]. These all are the potential causes of SIS and treatment of the condition widely depends upon its underlying etiology [16]. 

An abundant narrowing of the sub-acromial space and decreased acromio-humeral space take place during overhead activities at the shoulder and is widely known in patients with SIS, [17] which is common as external and primary impingement. Postural abnormalities and abnormal scapular motion could be the anatomical and biomechanical causes of SIS [18]. The exact endowment of the scapula leading to these impairments is still under study, whereas it has been claimed that an asymmetry in scapular protraction between the margins of both shoulders due to abnormal posture associated with SIS [19]. This could be due to other underlying disorders, such as upper cross syndrome, rotator cuff tears, bicipital tenosynovitis, or kyphosis, leading to abnormal posture and eventually causing SIS [20]. Some MRI studies have also confirmed that slightly increased shoulder protraction is one of the causes of reduced sub-acromial space [19, 21, 22]. A study contrasted it by affirming the association between abnormal scapular position and SIS [23]. The results of the study [23] showed no association between posture and shoulder overuse injuries when measured bi-dimensionally for scapular protraction. However, it was argued that measurements were 2-dimensional, which could limit the description of movement, whereas it was claimed that abnormal scapular movements become a risk factor for SIS [24]. Moreover, the scapular motion also plays an important role while moving the arm in the sagittal and frontal planes, and any impediment may be a cause of SIS [25]. Therefore, it is evident from the literature that the scapular range of motion should be considered when treating SIS.

Regarding treatment for SIS, a systematic review reported that there is low-quality evidence supporting the use of exercise therapy alone for SIS [26]. Hence, it signifies the importance of exercise therapy combined with other treatments such as manual therapy [26]. A trial reported significant and superior results when exercise therapy was combined with manual therapy for the treatment of SIS [3]. It has been demonstrated that exercise therapy improves the range of motion while manual therapy produces capsular extensibility [27]. Hence, a combination of both treatments tends to produce more efficacy than alone. Considering the discussed literature and physiological beliefs about the concept of combining exercise therapy and manual therapy appears to offer potentially favorable treatment results for SIS. The synergy between these two treatments, targets to induce extensibility and improve the range of motion within the shoulder joint [28]. 

Generally, increased scapular internal rotation and shoulder protraction with a decrease in shoulder upward rotation have been observed in patients with SIS [29]. It is suggested that therapeutic exercises might alter scapular control and in turn alleviate pain and improve function [2]. Many studies believe that strengthening the muscles around the shoulder along with stretching can effectively decrease pain and disability [1, 18, 21]. Nevertheless, various studies implied that the addition of manual therapy along with exercises could positively impact the rehabilitation of SIS to improve pain, range of motion and functional capacity [30, 31]. In contrast, some systematic reviews have found improved results while using exercise-based rehabilitation only [26, 32]. Therefore, keeping the conflicting results in mind, it is necessary to conduct further studies to investigate whether manual therapy or exercise therapy is effective in treating SIS.

A systematic approach to treating SIS is necessary, given its increasing prevalence in Pakistan [7, 8]. This study aimed to suffuse the clinical gaps by employing manual therapy for patients with SIS. The study would help clinicians, physiotherapists, and medical practitioners to devise an effective and less time-consuming treatment protocol for patients with SIS. The present study has made an effort to evaluate the efficacy of the addition of a manual therapy program in the treatment of SIS to effectively enhance treatment protocols. The purpose of this single-blind, randomized controlled trial (RCT) was to compare the effectiveness of both treatments. This study design may help to reduce bias and provide methodological rigour in examining the cause-effect relationship between two treatment groups [33]. This study aims to provide evidence-based practice towards the management of patients with SIS and treatment plans can be tailored according to each patient’s needs and condition. This study evaluated the effectiveness of strengthening and stretching exercises with and without manual therapy on functional capacity, pain, and scapular range of motion in patients with SIS. This study describes the impact of treatment on scapular ROM as a broader perspective for more comprehensive evaluation of shoulder biomechanics and function. Finally, it has been hypothesized that manual therapy along with exercise therapy would result in beneficial outcomes in patients other than those having exercise therapy alone.

## Methods

### Study design

This was a single-blinded randomized controlled trial conducted as per the guidelines of Helsinki [34]. Only the assessor was kept blinded due to the treatment regime. It is believed that while measuring the effectiveness of an intervention, it is difficult to blind patients and therapists while outcome assessor blinding can be achieved [35]. This research study was registered with the Iranian Registry of Clinical Trials ID (IRCT20230526058291N1). After approval from the institutional review board and the ethics committee of the University of Lahore with reference ID: REC-UOL-102-01-2022 dated 18/01/2022, the data collection process was started. Informed consent was obtained from the participants, and it was ensured that they would not undergo any treatment outside the trial, i.e., any other intervention or exercise at home, any medications, etc.

### Setting

The data were collected from the University of Lahore Teaching Hospital between March 2022 and December 2022.

### Sample size calculation

The sample size was derived from Sharma S. et al.’s study [3] through its outcome of pain measured through SPADI-H and was calculated by using the following formula:

$${N_1} = {\left\{ {{z_{1 - \alpha /2}}*\sqrt {\bar p*\bar q*(1 + \frac{1}{k})} + {z_{1 - \beta }}*\sqrt {{p_1}*{q_1} + (\frac{{{p_2}*{q_2}}}{k})} } \right\}^2}/{\Delta ^2}$$

Where Z 1 -α/2, i.e., type 1 error = 1.96, p1, p2 = estimated population (0.9, 0.48), q = 1-p, Z1-β, ι.ε., probability of type II error = 0.84, Δ = (p2-p1), i.e., absolute difference between two populations, and k = ratio of sample size for group 2 to group 1 = 1. The confidence interval was 95%, and the desired power was 0.8%. Based on this criterion, 32 participants were required in each group. Considering 10% attrition, data were collected from 36 participants. The sample was recruited through convenience sampling. A loss to follow-up of up to 20% was considered, for which 80% of the sample followed the treatment.

### Participant characteristics

Data were collected from both male and female patients with shoulder impingement syndrome aged from 25 to 40 years. SIS was diagnosed based on clinical presentation and examination or pre-diagnosis. Only those with chronic conditions of SIS, i.e., ≥ 3 months, were included. Participants with a history of non-traumatic onset of shoulder pain, pain during passive or isometric resisted external rotation of the arm at 90 degrees of abduction, pain with palpation of the rotator cuff tendons and 150 degrees of arm elevation, positive painful arc during active elevation of the arm, 1 or more positive SIS tests (Hawkins-Kennedy, Neer’s test) [36, 37]were included. The diagnostic accuracy for the applied clinical examination techniques with the sensitivity ranged between 0.69 and 0.78 while specificity was from 0.57 to 0.62, indicating these tests are useful to rule out SIS [36]. A moderate to substantial reliability was demonstrated for Hawkins Kennedy and Neer’s test from 0.45 to 0.67 [38]. It has been found that the clinical accuracy for the diagnosis of SIS can be achieved through a combination of tests [39]. Participants were excluded from the study if they had a history of clavicle, humerus, or scapular fracture and rotator cuff surgery, numbness or tingling of the upper limb that was reproduced by a cervical compression test, a positive sulcus or apprehension test, a positive drop arm test, systematic illness or corticosteroid injection within 3 months prior to intervention or physiotherapy within 6 months before intervention.

### Randomization and concealed allocation

A total of 32 participants were included in this trial, and utilizing computer-generated randomization, they were split into two treatment groups. However, 55 participants were analyzed first. Using Microsoft Excel, a computer-based random number generator, a simple randomization process was carried out. A sequence of 32 random numbers was created by the computer, and each participant was given a special identification number. Individuals served as the randomization unit. The use of computer-generated randomization guaranteed that the distribution of individuals among treatment groups was unbiased and equitable. Then, participants were divided into Group A or Group B using these random numbers. Participants in Group A received manual treatment along with stretching and strengthening exercises. Participants in Group B, on the other hand, were only given stretching and strengthening exercises; no manual therapy was provided.

Furthermore, the allocation was concealed using the sealed envelope method to prevent selection bias. This was ensured by opening envelopes from the first author of the study and assigning participants to one of the groups.

### Outcome measures

The outcomes and outcome measures for the study were as follows: the disability of the arm, shoulder, and hand (DASH)questionnaire was used to evaluate functional capacity, the numeric pain rating scale (NPRS) for pain, and goniometry was used for scapular ranges, i.e., scapular protraction and upwards rotation.

### Functional capacity

DASH is used to measure the functioning and disability of the upper limb. This is used to measure the patient’s ability to perform activities of daily living. This is a 30-item questionnaire in which the score ranges from 1 to 100, with the lowest score suggesting minimum disability and the highest score showing maximum disability [40]. The DASH demonstrated excellent reliability with an intra-class correlation coefficient (ICC_2, 1_) of 0.96 [41], validity (Pearson *r* > 0.70) and good responsiveness [42]. 

### Pain

Pain is measured by the NPRS, in which patients have been asked to rate their pain levels from 0 to 10; the higher the score is, the more severe the pain, i.e., within 7–10, moderate pain ranges from 4 to 6, and mild pain ranges from 0 to 3 [43]. The NPRS was tested on patients with shoulder pain to measure its reliability that was demonstrated as 0.74 indicating good ICC and was highly responsiveness [44]. 

### Scapular range of motion

The shoulder range of motion (ROM) involving scapular protraction and scapular upward motion was measured using a goniometer. The intrarater and interrater reliability for the goniometer was found as (ICC = 0.87 and 0.92) respectively, when measured for shoulder ROM indicating an excellent reliability with a minimal detectable change (MDC_95_) was 8° [45]. 

Following the present study, the scapular ranges were measured by asking the patient to sit comfortably and test the arm in a relaxed position at the side. The fulcrum of the goniometer was placed at the superior angle of the scapula by keeping the static arm at the midpoint of the thorax in the frontal plane. The moving arm was placed over the acromion process [46]. For protraction, the patient was then asked to gently initiate the movement to reach the arm in front of the body as in opening the door knob. For upward rotation, the patient was asked to move the arm above the head position as far as it was pain-free and easy to perform. The reference range for scapular protraction is 20°–30°, and for upwards rotation, it is 40°–50° [47]. The physiotherapist was well aware of any compensatory movement, i.e., flexing the trunk.

### Intervention group- exercise therapy + manual therapy

Group A was treated with exercises, and 45 min of manual therapy was given to each patient. The participants were assessed within a week before the intervention (baseline) and at the end of the 4-week intervention (follow-up). The patient received 3 sessions per week. Manual therapy was employed only on the involved side. Grade III and IV mobilization were performed, including arthro-kinematic movements for different sub-joints at the shoulder, such as the glenohumeral, scapulothoracic, sternoclavicular, and acromioclavicular joints, and cervical spine. The soft tissue techniques were also applied along with the contract/relax technique at the affected muscles. Previous literature supports the use of these techniques to treat SIS. Manual therapy was applied according to each patient’s presentation and needs. The progression of the treatment was dependent on the assessment at the time of each manual therapy session indicating any change in the intensity or frequency of treatment. The manual therapy involved glenohumeral joint mobilization (anterior, posterior, and inferior glides) with a rationale to reduce the resistance in movements and increase ROM. The anterior, posterior, and inferior glenohumeral joint mobilization increases external rotation, internal rotation, and abduction of the shoulder respectively [48]. Additionally, the long-axis distraction was also performed to address hypo-mobility. These mobilizations were performed at Grade I-II when there was pain up to 7–9 on the NPRS scale while Grade III-IV were performed in the cases where hypo-mobility and moderate pain up to 4–6 on the NPRS scale was found. Each mobilization was applied for the duration of 30 s with a rate of one mobilization every one to two seconds approximately, followed by a rest of 30 s. The frequency was a total of 5 sets of 30-second mobilisations each with a rest of 30 s in between. The manual therapy was performed by the physiotherapist with certified manual therapy training from Physio Connect Pakistan who has > 5 years of experience in the field of treating shoulder pathologies.

The exercise regime for Group A was the same as that for Group B described below.

### Control group- exercise therapy alone

Group B was given only strengthening and stretching exercises for both involved and uninvolved sides, and the session lasted for 25–30 min. All exercises were performed under the supervision of a physiotherapist with 5 years of clinical experience. The upper trapezius, pectoralis minor, and posterior part of the shoulder were targeted for the stretching and strengthening exercises as suggested by Camargo et al. [17] The exercise therapy involved 3 strengthening and 3 stretching exercises. For strengthening the muscles, exercises were performed with an external rotation of the shoulder, initiating with elbow flexion at 90° in the scapular plane. Participants were instructed to perform maximum external rotation in this position. Shoulder extension in the prone position was performed for the lower trapezius muscle. For pectoralis minor, wall push-ups were instructed to the participants. The maximum pain-free range of motion during all the exercises was ensured with the participants. The frequency of each exercise was 5 × 3 initially and progressed up to 5 × 5. The stretches were based on the finding of increased activation of pectoralis minor and upper trapezius muscles and posterior shoulder tightness having an association with shoulder pain and limited ROM. The 3 sets of exercises with 7 repetitions were completed with a rest of 30 s in between. The progression of exercises was managed in a pattern that included an increased number of repetitions after completion of the first week, i.e., 10 × 3. After the completion of two weeks, manual resistance was added that was increased up to the end of the treatment.

A detailed description of the intervention regime for both groups is presented in Table 1.

**Table 1:** Treatment regime for both groups

Group A Intervention group	Group B Comparison group
**1. Manual therapy** Grade III and IV mobilizations to shoulder sub-joints and cervical spine Soft tissue technique Contract/relax technique for the trapezius, serratus anterior, and pectoralis	**1. Stretching exercises**: Shoulder external and internal rotation stretch Shoulder rotations (backward and forwards) Shoulder flexion stretch
**2. Stretching exercises:** Shoulder external and internal rotation stretch Shoulder rotations (backward and forwards) Shoulder flexion stretch	**2. Strengthening exercises**: Wall push-ups Shoulder extension in prone lying External rotation with shoulder flexion at 90° Resisted shoulder protraction
**3. Strengthening exercises:** Wall push-ups Shoulder extension in prone lying External rotation with shoulder flexion at 90° Resisted shoulder protraction	

### Statistical analysis

SPSS for Windows software, version 21, was used to analyze the data using statistical significance *p* = 0.05. The Shapiro‒Wilk test was used to check the normality of the data, which was greater than 0.05, so the data were normal, and parametric tests of analysis were used. The frequencies and percentages are given for qualitative data. The quantitative data are presented as the mean and standard deviation. An independent sample t-test was employed to measure between-group changes.

## Results

**Fig. 1:**
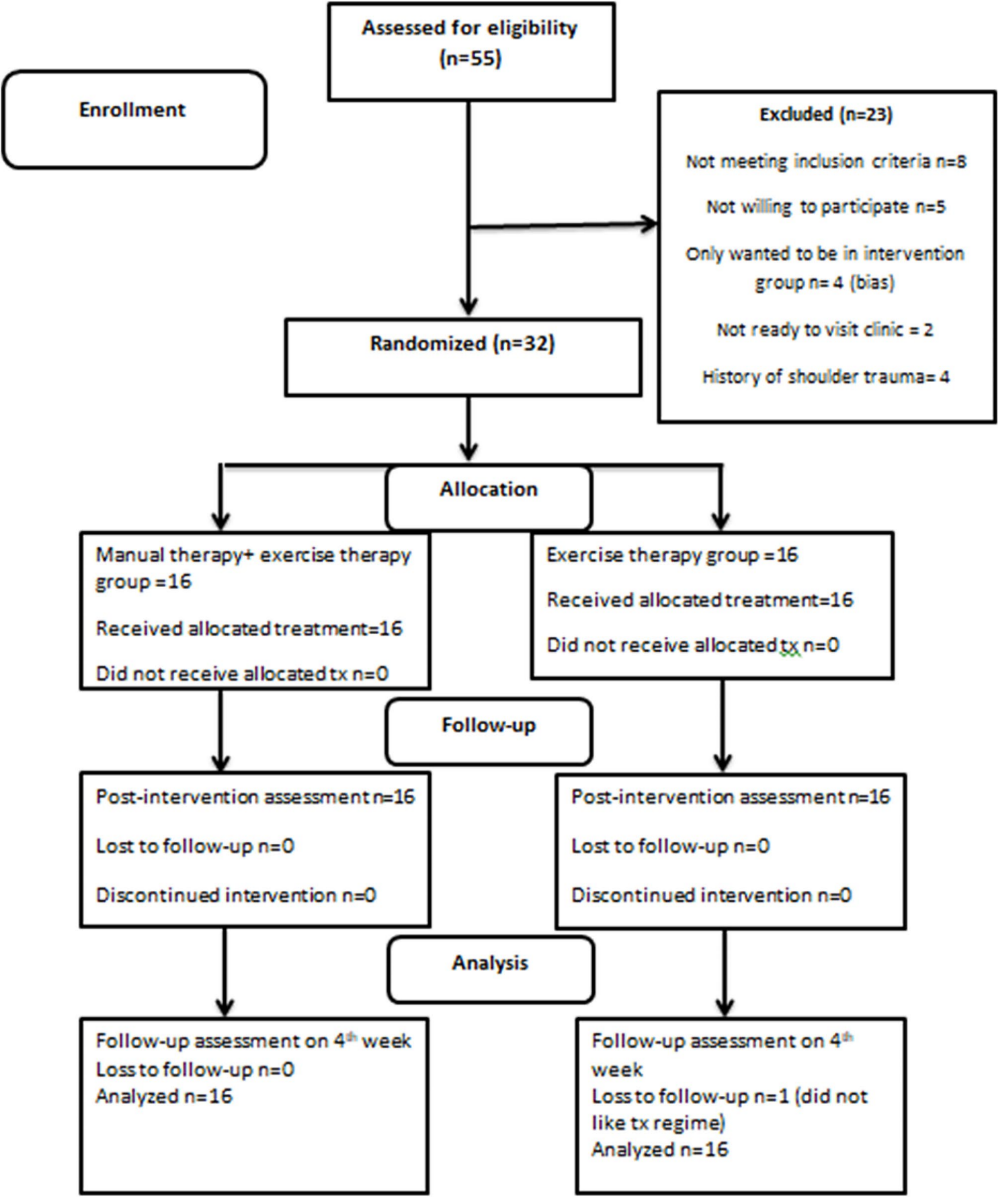
Consort Diagram for the description of the study

The demographic properties of the participants are presented in Table 2. The treatment undertook from March 2022 to December 2022. The study has followed the CONSORT guidelines [49]. Figure 1 shows the CONSORT diagram for the description of the study. Initially, 36 participants were randomized but before the treatment was processed, four participants did not want to be part of the treatment protocol. Hence, 32 participants were analyzed at the baseline and the follow-up. No drop-out was observed within the study once the treatment protocol started. There was no significant difference found in comparison. The mean age of the treatment group was 38.19 ± 7.31, while that of the comparison group was 35.69 ± 7.98. A total of 27 males and 35 females were recruited in the study and were randomly assigned to both groups. Baseline homogeneity between the two groups was not found, as shown in Table 2.

**Table 2:** Demographic characteristics

Variables		Exercise with manual therapy (*n* = 16)	Exercise Group (*n* = 16)	P value
		Mean ± SD	Mean ± SD	
Age (years)		38.19 ± 7.31	35.69 ± 7.98	0.23
**Gender**	Males Females	12 (37.5%) 20 (63.5%)	17(53.1%) 15 (46%)	0.33
**Health Status**	BMI	25.08 ± 6.28	23.23 ± 5.54	0.182
**Affected Shoulder**	Right Left	21 (65.6%) 11 (34.4%)	25 (78.1%) 07 (21.9%)	0.21
**Occupation**	Students Office workers Factory workers Housewives	02 (0.06%) 10 (31.2%) 07 (21.8%) 15 (46.9%)	00 (0.0%) 11 (34.3%) 10 (31.2%) 11 (34.3%)	

BMI = Body mass index = weight (kg)/height (m2)

There was no statistically significant difference when a between-group comparison was measured using an independent sample t-test at baseline. The pre-treatment values were recorded at the first visit of patients when they agreed to participate in the interventional study.

### Functional capacity

The DASH was used to measure functional capacity in which participants of both groups showed significant results, i.e., *p* < 0.05 after the treatment when measured at the final follow-up. There was not much difference in values between the groups. Within-group and between-group differences showed significant outcomes, as demonstrated in Table 3.

**Table 3:** Between Group and Within Group Changed scores for Shoulder Impingement syndrome for Functional Capacity (DASH), Pain (NPRS), and Shoulder Range of Motion (Scapular Protraction and Scapular Upward Rotation)

Variables	Measurements	Treatment group	Within group difference (Treatment group)	Control group	Within group difference (Control group)	P-value (Between group difference)
**Functional Capacity**	Baseline	26.13 ± 6.2	0.002	19.7 ± 4.32	0.012	0.01
	Follow-up	21.34 ± 4.5		16.25 ± 3.1		
**Pain**	Baseline	5.57 ± 1.46	0.03	6.04 ± 1.24	0.2*	0.001
	Follow-up	2.19 ± 1.05		4.63 ± 0.88		
**Scapular Protraction°**	Baseline	1.42 ± 1.36	0.05	2.43 ± 1.32	0.001	0.001
	Follow-up	11.25 ± 0.68		9.94 ± 0.072		
**Scapular Upward Rotation°**	Baseline	20.44 ± 3.23	0.001	10.63 ± 4.72	0.1*	0.001
	Follow-up	34.13 ± 2.78		24.63 ± 3.59		

SD = standard deviation; ROM = range of motion; NPRS = numeric pain rating scale; DASH = disabilities of arm, shoulder and hand

*=Insignificant i.e. >0.05

### Pain

The pain in the intervention group was 5.57 ± 1.46 at baseline, while it decreased up to 2.19 ± 1.05 after the treatment. Likewise, the pain score in the comparison group was 6.04 ± 1.24, and after exercise therapy, it was reduced to 4.63 ± 0.88. Both groups showed reduced values; however, a significant difference was found in the intervention group. Hence, the addition of manual therapy with the exercise group was more marked. According to between-group comparisons for pain, the score on the NPRS demonstrated the superiority of the experimental group.

### Scapular range of motion

SIS highly affects scapular protraction and upward rotation, which was significantly improved after the treatment in both groups and when compared between groups. Both groups reported a better range of motion for protraction, which was 1.42 ± 1.36 and 2.43 ± 1.32 before the treatment and improved up to 11.25 ± 0.68 and 9.94 ± 0.072 for groups A and B, respectively. However, group B showed a non-significant difference, i.e., *p* > 0.05, with upward rotation after the treatment, hence demonstrating no effect of exercise therapy alone on SIS. In contrast, the treatment group manifested significant results. Finally, when compared between groups using an independent sample t-test, the results were statistically significant, i.e., < 0.05, indicating the superiority of the intervention group.

Table 3 shows within group and between group differences for both treatment and control groups.

## Discussion

The purpose of this study was to assess the effects of exercise management with and without manual therapy on function, pain, and scapular movement in individuals with shoulder impingement syndrome. This study showed that the addition of manual therapy to an exercise protocol improved function, pain, and scapular movement after 4 weeks of intervention in individuals with SIS. This research emphasizes the importance of exercise with manual therapy in both male and female patients with shoulder impingement syndrome. The results indicated a significant improvement in functional capacity, pain, and scapular range of motion in the intervention group administered combination therapy i.e. exercise along with manual therapy. However, within-group analysis of the control group has shown statistically significant results for functional capacity and scapular protraction only. Thus, these results clarified that exercise with manual therapy is more efficient than exercise alone in terms of mentioned outcomes.

Various studies have been conducted to demonstrate different treatments for SIS [26, 50]. Therefore, it is quite difficult to determine which treatment might be effective for a particular patient. However, it is believed that exercise therapy is one must-have treatment in this condition, but to accelerate the rehabilitation process, manual therapy or other physiotherapy interventions have shown effectiveness [2]. Likewise, a randomized controlled trial was conducted in 2020 to demonstrate the effectiveness of exercise therapy along with neuromobilisation, which included sliding and tensioning of nerves [50]. The results were in favor of the group receiving both treatments compared to the group receiving exercise therapy alone. The study was of good quality, but some limitations can be noted; for example, only pain was measured, and there was no reporting of an improved range, which may put its external validity into question. Moreover, there was no blinding of patients and therapists which may increase the risk of measurement and selection bias. The blinding of the assessor was mentioned which was only for the allocation of patients; therefore it raises a concern that the assessment was not blinded. However, the authors have performed baseline homogeneity analysis and the p-value was found > 0.05 indicating successful randomization, thus control of selection bias can be seen. The study [50] only aimed to measure pain with intervention. The results were similar to those of this study, i.e., the pain was significantly decreased when measured with the NPRS. The NPRS is a reliable and valid measure, [51, 52] which allows for a quantitative and standardized assessment of pain intensity, making it convenient to analyze pain and compare outcomes across different studies [53]. The effect size for pain in the present study was 0.89, indicating a large effect of the treatment in comparison to the control group. Additionally, the age of the participants was almost the same as the age of participants in the present study. Therefore, the results of the study can be found relevant.

Considering the pain and functional capacity in the present study, within-group analysis of both groups showed significant results for functional capacity. However, pain was only found significant in the intervention group. This suggests that both groups are not comparable in terms of pain and hence, claimed additional benefits of manual therapy. Moreover, the treatment group showed a minimal detectable change of 4.8 points and affirmed a greater clinical improvement. However, improved functional capacity in the control group can be justified by exercise therapy comprising stretching and strengthening in both groups. One of the other reasons for the achieved functional capacity is that the DASH score was already at a minimum at the baseline measurement. Hence, an already decreased score can be assumed to be an improvement in the control group. These results are similar to those reported following an RCT by Alexa et al. [54], where exercise therapy has shown clinically and statistically improved results. It can be debated that only the sports population was included in the study [54] whereas the present study included the general population. The results of the present study support the statistical and clinical improvement of the addition of manual therapy with exercise therapy. However, clinical improvement in the control group cannot be overlooked. Manual therapy has been proven effective previously when compared with other treatments such as exercises, upper thoracic or posterior shoulder interventions, and injection therapy [3, 55, 56], which is quite similar to the results of the present study implying the superiority of manual therapy.

Altered scapular kinematics has been observed in many studies due to shoulder impingement syndrome [57–59]. Hypothetically, a decrease in scapular external rotation, upward rotation and sometimes protraction may reduce the sub-acromial space, leading to impingement at the shoulder [57]. However, previous literature shows discrepancies in reporting shoulder kinematics in patients with SIS; for example, many studies stated decreased shoulder upwards rotation, [24, 60] while a study claimed an increase in upwards rotation, [61] whereas there are studies that believed that there was no movement alteration at the scapula due to SIS [17]. A study back in 2012 was conducted specifically to analyze scapular motions during SIS and found that scapular movements are altered during SIS [62]. This was also confirmed by further studies that stated altered biomechanics at the shoulder joint [63, 64]. Keeping these results in mind, the present study measured the scapular ranges at baseline and the final follow-up; the results were that scapular ranges improved after the treatment, leading to pain-free movement in the involved arm.

A systematic review in 2020 highlighted the fact that improvement in scapular ranges by applying exercise and manual therapy together can be due to manual therapy having greater effects on muscular pain and soreness [65]. A systematic review is believed to have the most reliable and valid findings, [66] thus results of the study [65] make high impact. This improvement in scapular ranges may be due to the breakdown of adhesions and maintenance of joint nutrition due to a better supply of blood after the application of exercise and manual therapy together [67]. Manual therapy not only decreases pain but also helps improve functional development and stretch the shortened tissues around and inside the joint while improving blood circulation [67, 68]. Sharma S. et al. in 2021 found that when manual therapy was combined with exercise therapy, the results were superior in this group [3]. However, a study in 2015 contrasted the results by claiming that the addition of manual therapy does not affect the treatment of SIS, [4] whereas the present study supports the use of manual therapy along with exercise therapy. A closer examination of this study indicated that the stage of impingement was not specified, which led to ambiguity, as acute, sub-acute, and chronic stages may alter the treatment duration [17]. Similarly, the present study also found statistical and clinical improvement in the range of motion at the shoulder joint with the addition of manual therapy with exercise therapy however, it could be noted that the present study recruited only patients with the chronic phase of SIS. Therefore, the results should be interpreted with caution for acute patients of SIS. A meta-analysis in 2017 showed that the addition of any other physiotherapy intervention, either electrotherapy, laser therapy, or manual therapy, is effective for the treatment of SIS [26]. Therefore, it implies the use of additional therapy to boot exercise therapy that is effective in treating shoulder impingement.

A recent randomized controlled trial in 2023 reported no effects of including scapular mobilization in the treatment protocol for subacromial impingement syndrome [69]. The study suggested employing exercise therapy as the primary intervention for this condition. Notably, the study was of good quality on the PEDro scale i.e. 8/10. The results of the study were in contrast with the findings of the present study, it could be argued that Hector et al. (2023) prescribed oral naproxen 500 mg twice daily for continuous 2 weeks, which is an NSAID used to alleviate the pain [70, 71]. Hence, the oral absorption of pain relieving medicine can mitigate the effects of physiotherapy i.e. scapular mobilizations as well as exercise therapy. Moreover, participants in the control group which showed improved outcomes for pain and functional capacity have already reported reduced pain at the baseline. Therefore, the results of the study can be ambiguous for its external validity. Nonetheless, the present study has set the criteria not to include participants having any other treatment such as pharmacological treatment. Therefore, the present study provides full insights into manual therapy and exercise therapy by limiting the confounders. Furthermore, the present study involved manual therapy for the shoulder as well as the cervical region which could be the reason for improved outcomes.

The present study can help clinicians add manual therapy relentlessly in the treatment of SIS, as the literature is in favour of adding an intervention to exercise therapy. Therefore, adding manual therapy will show earlier results, and effectiveness can be measured to improve pain, disability, and scapular ranges. The measurement of scapular ROM adds novelty to the study, by evaluating the impact of intervention on the coordinated movement of the shoulder girdle. Moreover, this study would demonstrate updated evidence for the treatment of SIS.

The limitation of the study was data were collected from only one setting and the sample size was relatively small, which could impact the generalizability of the study and a threat to its external validity. The results should be interpreted with caution for patients in acute conditions, as the study was conducted on chronic cases of SIS. One of the limitations was that the exact cause of SIS in patients was not investigated. There should be more follow-ups within the study to obtain more clear insight. Hence, keeping in consideration, multi-center studies with larger sample size are recommended to conduct in the future. Further, it can be argued that not blinding the physiotherapists may cause bias in the results. However, they did not collect the data directly; they just applied the treatment, and only the assessor collected the data without the role of the treating physiotherapist. Additionally, the physiotherapists were also unaware of which group they had been assigned. Moreover, it could be noticed that the experimental group received more treatment in terms of duration than the control group which may affect the outcomes that may act as confounder. However, there were no harms of each treatment observed in any group. Likewise, the study has employed standardized data measurement tools to limit the confounders and minimise bias which may ensure reliability and validity of study results.

## Conclusion

The findings of the study suggest improvements in functional capacity, pain, and shoulder range of motion after the addition of manual therapy with exercise therapy in patients with shoulder impingement syndrome. The addition of manual therapy showed superior effects than exercise therapy alone for the chronic condition of SIS. Further studies are required to observe the long-term effects of the treatment protocol.

### Electronic supplementary material

Below is the link to the electronic supplementary material.

Supplementary Material 1

## Data Availability

All data generated or analysed during this study are included in this published article [and its supplementary information files].

## Abbreviations

CONSORT: Consolidated Standards of Reporting Trials
BMI: Body Mass Index
SIS: Shoulder Impingement Syndrome
ROM: Range of Motion
NPRS: Numeric Pain Rating Scale
Dash: Disability of Arm Shoulder and Hand
PEDro: Physiotherapy Evidence Database
SPADI-H: Shoulder Pain and disability index-Hindi
