# Supplementary material for: Effects of manual therapy in addition to stretching and strengthening exercises to improve scapular range of motion, functional capacity and pain in patients with shoulder impingement syndrome: a randomized controlled trial
Source: BMC Musculoskelet Disord. 2024 Mar 2;25:192. doi: 10.1186/s12891-024-07294-4 (PMC10908164; doi:10.1186/s12891-024-07294-4)
Supplement: Supplementary file 1 — Supplementary Material 1 [file 12891_2024_7294_MOESM1_ESM.docx]

**Table S1: Between group differences**

| **Variables** | **Intervention group** | | **p-value** | **Control group** | | **p-value** |
| --- | --- | --- | --- | --- | --- | --- |
|  | **Pre-treatment** | **Post-treatment** |  | **Pre-treatment** | **Post-treatment** |  |
| DASH | 26.13±6.2 | 21.34±4.5 | 0.002 | 19.7±4.32 | 16.25±3.1 | 0.012 |
| NPRS | 5.57 ± 1.46 | 2.19±1.05 | 0.03 | 6.04 ± 1.24 | 4.63±0.88 | 0.2 |
| Scapular Protraction° | 1.42± 1.36 | 11.25± 0.68 | 0.05 | 2.43± 1.32 | 9.94± 0.072 | 0.001 |
| Scapular Upwards Rotation° | 20.44 ±3.23 | 34.13 ±2.78 | 0.001 | 10.63 ±4.72 | 24.63±3.59 | 0.1 |

SD=standard deviation; ROM=range of motion; NPRS=numeric pain rating scale; DASH=disabilities of arm, shoulder and hand

**Table S2: Difference within groups**

| **Variables** | | **Treatment groups (Difference within groups)** | | **p-value** |
| --- | --- | --- | --- | --- |
|  |  | **Manual therapy with exercise**  **Mean ± SD** | **Exercise therapy group**  **Mean ± SD** |  |
| DASH | | 4.79±1.7 | 3.45±1.22 | 0.01 |
| NPRS | | 3.38±0.49 | 1.41±0.36 | 0.001 |
| **Scapular ROM** | Scapular Protraction° | -9.83± 0.95 | -7.51± 1.248 | 0.001 |
|  | Scapular Upward Rotation° | -13.69 ±0.45 | -14±1.13 | 0.001 |

SD=standard Deviation; ROM=Range of motion; NPRS=Numeric Pain Rating Scale; DASH=Disabilities of arm, shoulder and hand
